# Development and testing of a self administered version of the Freezing of Gait Questionnaire

**DOI:** 10.1186/1471-2377-10-85

**Published:** 2010-09-23

**Authors:** Maria H Nilsson, Gun-Marie Hariz, Klas Wictorin, Michael Miller, Lars Forsgren, Peter Hagell

**Affiliations:** 1 Department of Health Sciences, Lund University, Lund, Sweden; 2 Department of Clinical Sciences, Division of Neurosurgery, Skåne University Hospital, Lund, Sweden; 3 Department of Community Medicine and Rehabilitation, Umeå University, Umeå, Sweden; 4 Department of Pharmacology and Clinical Neuroscience, Umeå University, Umeå, Sweden; 5 Department of Clinical Sciences, Division of Neurology, Skåne University Hospital, Lund, Sweden

## Abstract

**Background:**

The Freezing of Gait Questionnaire (FOGQ) was developed in response to the difficulties of observing and quantifying freezing of gait (FOG) clinically as well as in laboratory settings. However, as the FOGQ is a clinician-administered patient-reported rating scale it cannot be used in postal surveys. Here we report the development and measurement properties of a self-administered version of the FOGQ (FOGQsa).

**Methods:**

A clinical sample and a postal survey sample of non-demented people with Parkinson's disease (PD; total n = 225) completed the FOGQsa and questionnaires concerning physical functioning (PF) and fall-related self efficacy (FES). Additional questions (No/Yes) regarded previous falls and whether they were afraid of falling. The clinical sample was also assessed with the Unified PD Rating Scale (UPDRS). Thirty-five participants completed FOGQsa and were also assessed with the original version (FOGQ) in a clinical interview.

**Results:**

There were no differences (P = 0.12) between FOGQ (median, 10; q1-q3, 2-14) and FOGQsa (median, 8; 2-14) scores. The Spearman (r_s_) and intra-class correlations between the two were 0.92 and 0.91 (95% CI, 0.82-0.95), respectively. For FOGQsa, corrected item-total correlations ranged between 0.68-0.89. Reliability was 0.93 (95% CI, 0.91-0.94). FOGQsa scores correlated strongest with UPDRS Item 14 (Freezing; r_s_, 0.76) and with FES (r_s_, -0.74). The weakest correlation was found with age (r_s_, 0.14). Fallers scored significantly (p < 0.001) higher on FOGQsa compared to non-fallers, median scores 8 (q1-q3, 4-14) versus 2 (0-7). Those expressing a fear of falling scored higher (p < 0.001) than those who did not, median scores 2 (0-7) versus 6 (2-14).

**Conclusions:**

The present findings indicate that the FOGQsa is as reliable and valid as the original interview administered FOGQ version. This has important clinical implications when investigating FOG in large scale studies.

## Background

Up to 50% of people with Parkinson's disease (PD) experience sudden and transient motor blocks (freezing) while initiating or performing activities [1]. Freezing of gait (FOG) is often described as if the feet are glued to the ground [1,2]. This description is recommended to use when asking people with PD about the phenomenon [3]. FOG typically appears when initiating the first step (start hesitation), on turning or when approaching a target (destination hesitation or "target freezing") [1,2]. FOG is usually evoked in crowded and confined spaces as well as in time limiting situations, e.g., when crossing a street [1,2,4-7].

The presence and severity of FOG has been found to be associated with longer disease duration [1,7,8], more advanced disease stage [1,7,8], falls [6,9-13], dyskinesias [1] and decreased mobility [14]. FOG is in fact one of the most distressing symptoms in PD [15], and it has a negative impact on patient perceived health [14,16].

FOG is characterized by its unpredictable and episodic nature. It typically occurs as short lasting episodes at home, which makes it difficult to observe and quantify during clinical testing as well as in laboratory settings [17,18]. Evaluating FOG is commonly done by either posing a question or by using a single item assessment such as item 14 of the Unified PD Rating Scale (UPDRS) [8-10,13]. Therefore, Giladi et al. developed the Freezing of Gait Questionnaire (FOGQ) which is a clinician/interview administered patient-reported rating scale [19]. The FOGQ consists of six items scored from 0-4. The total score ranges from 0 to 24, and higher scores denote more severe FOG.

The FOGQ was originally tested for reliability (Cronbach's alpha, 0.94) and validity in 40 people with PD [19]. However, although the FOGQ appears to be a useful and valid tool for its purpose, the dependency on a clinician to interview the patient and to demonstrate the meaning of FOG is also limiting. For example, the tool becomes somewhat time consuming for implementation in clinical practice and cannot be used for larger scale postal surveys. A completely self-administered FOGQ scale that provides the same type of information would therefore be of great benefit.

This study assessed the validity and reliability of a self-administered version of the FOGQ, i.e. the FOGQsa. In addition, we explored the relationships between FOGQsa scores and patient demographics, fear of falling, falls, gait, motor complications and physical functioning.

## Methods

The study was divided into two phases: (1) development of a self-administered version of FOGQ (the FOGQsa) and (2) testing the psychometric properties of the FOGQsa.

### Development of the FOGQsa

The intention was to preserve the semantic content of all six original FOGQ items but with revisions of the wording to make them applicable for self-administration. In accordance with the original FOGQ instrument [19], responses should be based on experiences over the last week except for item 3. As in the original FOGQ scale, all items are scored from 0-4 (possible total score range, 0-24; higher scores = more severe FOG).

To substitute for the demonstration and description of FOG in the FOGQ, a cover sheet explaining the FOG phenomenon was developed for the FOGQsa. The description was based on a review of the literature, and it was constructed in close interaction with two nurses and two neurologists specialized in PD. The cover sheet describes that FOG is typically experienced as if "the feet are glued to the ground", and that this can happen while trying to initiate walking, while walking (which could be accompanied by taking smaller and smaller steps) or while turning. Other provocative factors are also described such as walking in confined spaces (e.g. passing a doorway), target hesitation (e.g. just before approaching a chair when sitting down) and when having limited time (e.g. crossing a street) [1,2,4-7].

Following modification of the FOGQ into the FOGQsa, face-to-face field-test interviews were conducted with 14 non-demented people with PD (9 men) with varying degrees of motor complications and/or gait disturbances to assess clarity of wording, face validity and respondent burden.

### Psychometric properties of the FOGQsa

#### Participants

Data collection included a clinical sample and a postal survey sample of non-demented people with idiopathic PD [20].

The clinical sample consisted of a convenience sample with varying degrees of motor complications and/or gait disturbances receiving out-patient care at a Swedish university hospital (Table 1). Fifty patients were invited to participate. Eight patients declined participation and five patients were unable to attend the study appointment. An additional participant did not complete the FOGQsa and was therefore excluded. Thus, 36 participants were included.

**Table 1:** Participants' characteristics

	Total sample n = 225	Clinical sample n = 36	Postal survey n = 189
Mean age, years (SD, min.-max.)	69.1 (8.9)	65.3 (6.2)	69.9 (9.1)
	42.0-91.0	49.0-83.0	42.0-91.0
	n = 221	n = 36	n = 185
Mean PD-duration, years (SD, min.-max.)	7.7 (6.8)	14.6 (8.3)	6.2 (5.5)
	0.33-44.0	2.2-44.0	0.33-28.0
	n = 210	n = 36	n = 174
Gender (men/women), n (%)	137 (62%)/	29 (81%)/	108 (58%)/
	84 (38%)	7 (19%)	77 (42%)
	n = 221	n = 36	n= 185
Falls past 6 months (yes/no), n (%)	88 (40%)/	19 (53%)/	69 (38%)/
	131 (60%)	17 (47%)	114 (62%)
	n = 219	n = 36	n = 183
Fear of falling (yes/no), n (%)	94 (43%)/	9 (25%)/	85 (46%)/
	125 (57%)	27 (75%)	98 (54%)
	n = 219	n = 36	n = 183
	Median, q1-q3	Median, q1-q3	Median, q1-q3
Falls-Efficacy Scale (S)	112.0	106.5	114.0
	75.0-130.0	94.8-129.0	69.0-130.0
	n = 183	n = 30	n = 153
Physical Functioning (PF, SF-36)	55.0	24.0	60.0
	29.0-75.0	20.0-28.0	35.0-80.0
	n = 218	n = 30	n = 188
Item 13, UPDRS II (Falling)	0.0	0.0	0.0
	0.0-1.0	0.0-1.0	0.0-1.0
	n = 223	n = 36	n = 187
Item 14, UPDRS II (Freezing)	0.0	0.0	0.0
	0.0-1.0	0.0-1.8	0.0-1.0
	n = 221	n = 36	n = 185
Item 15, UPDRS II (Walking)	1.0	1.0	1.0
	0.0-2.0	0.0-1.0	0.0-2.0
	n = 221	n = 34	n = 187

Falls-Efficacy Scale, Swedish version; the total score can range between 0-130 (higher scores = better).

Physical Functioning (PF, SF-36); total score range between 0-100 (higher scores = better).

Item 13, 14 and 15 of the UPDRS part II; each item graded from 0-4 (higher scores = greater severity).

Additional data regarding the participants are presented in Table 3.

The postal survey sample was recruited from another Swedish university hospital (Table 1). The survey was sent to 282 patients (39% female), and 231 were returned, of which 191 were completed (43% female; conservative total response rate, 68%). Thirty-eight questionnaires were returned completely blank and two were returned to sender due to change of address. Out of the 191 responders, two had left all items of the FOGQsa blank. These two participants were excluded, leaving 189 participants for analyses. Demographic data of all 225 participants are presented in Table 1.

#### Instruments

All participants completed the FOGQsa, the Swedish version of the Falls-Efficacy Scale, FES(S) [21], and the Physical Functioning (PF) scale of the Medical Outcomes Study 36-Item Short-Form Health Survey (SF-36) [22].

FES(S) assesses fall-related self-efficacy, and the 13 activities are rated from 0 (not confident at all) to 10 (completely confident) [21]. The total score can range from 0 to 130. PF comprises ten items about physical activities [22]. The score can range from 0 to 100, where higher scores denote better physical functioning. Additional questions included whether participants had fallen during the past six months (No/Yes) and if they were afraid of falling (No/Yes). The clinical sample was also asked whether they felt unsteady while turning (No/Yes).

#### Procedure

The FOGQsa was mailed to the clinical sample before the study visit. The participants were instructed to complete it three days before the visit, put the completed questionnaire in an envelope, seal it and bring it to the study visit. Demographic data (Table 1) were collected through self report for all participants, which the clinical sample completed at their out-patient visit. Demographic questions preceded the included self-administered questionnaires. In addition, the clinical sample was assessed with timed gait tests: ten meter walk test (gait speed, both comfortable and fast) and Timed Up & Go (TUG). Each gait test had one practice trial and one test trial. The FOGQ was thereafter administered as a clinical interview including a demonstration of FOG [19]. That is, the assessor demonstrated typical manifestations of start hesitation, freezing at the doorway and while turning. Finally, clinical assessments according to the UPDRS and Hoehn & Yahr stages (HY) [23] were conducted by an independent assessor who was unaware of previous test results. HY was also assessed for the "off" phase based on patient-reported history. In the postal survey sample, self administered versions of UPDRS items 13 (falling), 14 (freezing) and 15 (walking) were included [24]. All participants of the postal survey sample received a reminder about ten days after the first administration.

The study was reviewed by the local ethics advisory committee, and the included participants gave their written informed consent.

##### Analyses

The primary aim of the analyses was to explore to what extent the FOGQsa scores reproduced the findings of the original FOGQ instrument [7,19,25].

We thus explored whether the assumptions for summing FOGQsa item scores into a total score were met [26]. That is, we examined whether item mean scores and standard deviations were roughly similar and if the corrected item-total correlations exceeded 0.3. Whether the items appear to represent a common variable was considered supported if corrected item-total correlations were ≥0.4 [26].

Floor and ceiling effects (i.e., the proportion of people obtaining minimum and maximum scores, respectively) were examined, and should be <15-20% [27,28]. Reliability (Cronbach's alpha) was also estimated, and should be ≥0.8 [29]. In addition, the standard error of measurement (SEM) was estimated using the formula:

SEM=SD×√(1-α)

Using data from the clinical sample, we assessed the relationship between the original FOGQ and the FOGQsa through Spearman (r_S_) and intra-class correlations (ICC; one-way random model). The correlations were expected to be strong (>0.8) in order to support criterion-related validity. Construct validity was assessed by examining the pattern of correlations (r_S_) between FOGQsa and UPDRS scores. To replicate the patterns reported for the original version of FOGQ [19,25], the correlation with FOGQsa scores was expected to be strongest for UPDRS part II (Activities of Daily Living, ADL) and weakest for UPDRS part I (mentation, behavior and mood) [7,19,25]. Among individual UPDRS items, we expected a stronger correlation between FOGQsa scores and item 14 (freezing) than with items 13 (falling unrelated to freezing), 15 (walking), 29 (gait) and 30 (postural stability) [19,25]. The correlation with disease severity (HY) was expected to be stronger in the "off" state than in the "on" state [25].

In addition, we examined the relationships between FOGQsa scores and patient demographics, FES(S), timed gait tests, dyskinesias (items 32-35 of the UPDRS part IV), motor fluctuations (items 36-39 of the UPDRS part IV) and PF. We expected the FOGQsa scores to correlate stronger with dyskinesia, motor fluctuations and FES(S) scores than with gait tests [25]. The weakest correlation was expected with age [25]. We also assessed differences (Mann-Whitney U test) in FOGQsa scores between people who did and did not report falls, fear of falling and unsteadiness while turning. It was expected that fallers, those reporting fear of falling and those being unsteady while turning would score higher.

Analyses were performed using SPSS 15 (SPSS Inc., Chicago, IL) and ScoreRelCI (Barnette 2005). Two-tailed p-values <0.05 were considered statistically significant. P-values were not corrected for multiple testing.

## Results

Field-test participants had a mean age of 64 (SD, 5.2) years and an average PD-duration of 16 (SD, 5.5) years. All 14 participants found the description of FOG to be clear and understandable and items were considered relevant and easy to answer. One participant commented that the duration of freezing was somewhat difficult to estimate. Two comments related to the frequency of freezing. However, no changes in the questionnaire were deemed necessary due to these comments. The mean time for completing FOGQsa was 3 (SD, 1.3) minutes.

For the total sample, mean and SD for FOGQsa items scores ranged between 0.76-1.6 and 1.1-1.4, respectively (Table 2). Corrected item-total correlations ranged between 0.68-0.89. Forty-one participants (19%) scored 0, and none reached the maximum score of 24. Reliability was 0.93 (95% CI, 0.91-0.94), and SEM was 1.6. Results for the two subsamples were similar (Table 2). Total FOGQsa scores could be computed for 214 (95%) out of the 225 participants.

**Table 2:** Descriptive and psychometric FOGQsa data

		Total sample n = 225		Clinical sample n = 36		Postal survey sample n = 189	
**Item**		**Mean (SD)**	***Corrected Item-total correlations *n = 214**	**Mean (SD)**	***Corrected Item-total correlations *n = 35**	**Mean (SD)**	***Corrected Item-total correlations *n = 179**

**1**	"Walking during worst state"	1.6 (1.1) 2 missing	0.68	1.7 (1.2) 1 missing	0.87	1.6 (1.1) 1 missing	0.64
**2**	"Gait difficulties affecting daily activities and independence"	1.1 (1.1) 2 missing	0.68	1.2 (1.1) 1 missing	0.76	1.1 (1.2) 1 missing	0.68
**3**	"Feet getting glued to the floor"	1.1 (1.4) ^1^ 3 missing	0.83	1.7 (1.4)	0.82	1.0 (1.4) 2 missing	0.84
**4**	"Duration of longest freezing episode"	0.87 (1.3) 7 missing	0.89	1.6 (1.6)	0.91	0.73 (1.1) 7 missing	0.90
**5**	"Duration of typical start hesitation"	0.76 (1.1) 6 missing	0.88	1.2 (1.4)	0.85	0.67 (1.1) 6 missing	0.89
**6**	"Duration of typical turning hesitation"	0.76 (1.1) 5 missing	0.83	1.1 (1.4)	0.78	0.68 (1.1) 5 missing	0.85

		Total score n = 214		Total score n = 35		Total score n = 179	

	Mean (SD)	6.1 (6.2)		8.6 (7.2)		5.6 (5.9)	
	Median, q1-q3	4.0, 1.0-4.0		8.0, 2.0-14.0		4.0, 1.0-9.0	
	Min-max	0-22		0-22		0-22	
	% scoring minimum (0)/maximum (24)	19/0		17/0		20/0	
	Reliability, Cronbach's alpha (95% CI)	0.93 (0.91-0.94)		0.94 (0.90-0.97)		0.93 (0.91-0.94)	

FOGQsa: Freezing of Gait Questionnaire, self administered version. Each item is scored between 0-4, and the total score thus ranges between 0-24. Higher scores denote more severe FOG.

Some participants left certain items blank. Total scores could therefore be computed for 214 out of the 225 (95%).

^1^ On item 3, 126 scored 0, i.e. they never experienced freezing episodes, whereas 96 out of the 214 (45%) scored at least 1 and could be defined as "freezers".

Out of the 96 reporting freezing episodes, 51 did so "often- about once a day" and 11 "always- whenever walking".

**Figure 1:**
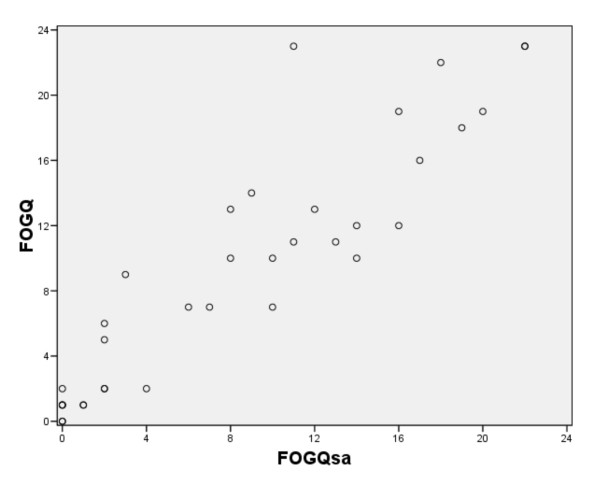
**The relationship between scores of the new self administered version of the Freezing of gait (FOG) questionnaire (FOGQsa) and the original interview administered FOGQ (n = 35)**.

Data for both the FOGQsa and the original FOGQ were available for 35 people in the clinical sample. There were no differences (P = 0.12) between FOGQ (median, 10; q1-q3, 2-14) and FOGQsa (median, 8; q1-q3, 2-14) scores. The Spearman and intra-class correlations were 0.92 and 0.91 (95% CI, 0.82-0.95), respectively (Figure 1). The mean number of days between completions was 2.6 (SD, 0.80).

The relationships between FOGQsa scores and other variables are presented in Table 3. The FOGQsa scores for the total sample showed the strongest correlation with UPDRS item 14 (Freezing; r_s_, 0.76) and with FES(S) (r_s_, -0.74). The weakest correlation was found with age (r_s_, 0.14). For common variables, both samples showed similar patterns (Table 3). However, the clinical sample showed a stronger correlation with UPDRS part II (r_s_, 0.68) than with FES(S) (r_s_, -0.57) (Table 3).

**Table 3:** Correlations with FOGQsa and descriptive values for clinical tests

	Total sample n = 225		Clinical sample n = 36		Postal survey n = 189	
	r_s_	p-value	r_s_	p-value	r_s_	p-value
Age, years	0.14	0.046	0.07	>0.3	0.21	0.006
PD-duration, years	0.42	<0.001	0.50	0.002	0.40	<0.001
Falls-Efficacy Scale	-0.74	<0.001	-0.57	0.001	-0.78	<0.001
Physical Functioning (PF, SF-36)	-0.61	<0.001	-0.50	0.006	-0.68	<0.001
Item 13, UPDRS II (Falling)	0.49	<0.001	0.53	0.001	0.49	<0.001
Item 14, UPDRS II (Freezing)	0.76	<0.001	0.72	<0.001	0.78	<0.001
Item 15, UPDRS II (Walking)	0.57	<0.001	0.56	0.001	0.64	<0.001

*Clinical sample*, n = 36	Median (q1-q3)		r_s_	p-value		

UPDRS I, Mentation, behavior and mood	2.0 (0-3.0)		0.33	0.059		
UPDRS II, Activities of Daily Living	7 (3.0-13.5)		0.68	<0.001		
UPDRS III, Motor examination	15.0 (8.0-20.0)		0.55	0.001		
Item 29, UPDRS III (Gait)	0.5 (0-1.0)		0.46	0.006		
Item 30, UPDRS III (Postural stability)	1.0 (0-2.0)		0.49	0.003		
Hoehn and Yahr ("on")	3.0 (2.0-3.0)		0.44	0.009		
Hoehn and Yahr ("off")	3.0 (3.0-4.0)		0.58	<0.001		
Dyskinesias (UPDRS IV, items 32-35)	2.5 (1.0-5.0)		0.63	<0.001		
Motor fluctuations (UPDRS IV, items 36-39)	3.0 (1.3-3.8)		0.60	<0.001		
Timed Up & Go, sec (Mean, SD)	9.7 (2.7)		0.34	0.049		
Comfortable gait speed, m/s (Mean, SD)	1.32 (0.23)		-0.29	0.094		
Fast gait speed, m/s (Mean, SD)	1.60 (0.40)		-0.33	0.055		

Falls-Efficacy Scale, Swedish version; the total score can range between 0-130 (higher scores = better).

Physical Functioning (PF, SF-36); total score can range between 0-100 (higher scores = better).

Item 13, 14 and 15 of the UPDRS part II; each item graded from 0-4 (higher scores = greater severity).

UPDRS part I; total score range 0-16 (16 = greater severity). UPDRS part II; total score range 0-52 (52 = greater severity). UPDRS part III; total score range 0-108 (108 = greater severity).

Dyskinesias (UPDRS part IV, items 32-35); total score range 0-13 (13 = greater severity).

Motor fluctuations (UPDRS part IV, items 36-39); total score range 0-7 (7 = greater severity).

FOGQsa scores did not differ (p > 0.3) between genders. For the total sample, the median score was 4 for both women and men (q1-q3, 1-9 and 1-11, respectively). Out of the 225 participants, 219 responded to the question whether they had fallen during the past six months. Fallers (88/219; 40%) scored significantly (p < 0.001) higher on FOGQsa compared to non-fallers, median scores 8 (q1-q3, 4-14) versus 2 (q1-q3, 0-7). Those expressing a fear of falling scored higher (p < 0.001) than those who did not, median scores 2 (q1-q3, 0-7) versus 6 (q1-q3, 2-14). In the clinical sample, those reporting unsteadiness while turning (20/35; 57%) had significantly (p = 0.030) higher FOGQsa scores than those who did not, median scores 11 (q1-q3, 6-16) versus 2 (0-13).

## Discussion

The present findings indicate that the FOGQsa is as reliable and valid as the original interview administered FOGQ version. Specifically, our observations support the legitimacy of summing item scores into a reliable total score that corresponds closely to FOGQ scores. In addition, construct validity is supported by a correlation pattern very similar to that reported for the original FOGQ version. This has important clinical implications when investigating FOG.

Snijders et al. highlighted that the original FOGQ does not include provocative circumstances of FOG [3]. When using a self-administered assessment it is crucial that the investigated construct is well defined and described. The cover sheet of the FOGQsa was created in order to make certain that the individual recognizes and fully understands the investigated phenomenon. It therefore included both environmental and circumstantial factors that may provoke FOG; narrow space (e.g. passing a doorway) [1,2,6,7], crowded space and limited time, e.g. trying to cross a busy street [2,4-6]. The original FOGQ does not mention target hesitation although FOG is known to occur when reaching a target [1,2,4,5]. This new self administered version does, however, include target hesitation when describing FOG. Field testing of the FOGQsa revealed that the participants considered the content to be both clear and relevant. The respondent burden was marginal as indicated by a short completion time.

FOGQsa had an internal consistency reliability above 0.90 and thus exceeded the recommendation of 0.80 [29]. This finding is similar to the results for the original FOGQ version [7,19,25]. In a separate pilot study, we have also found its test-retest reliability to be adequate (ICC, 0.85) [30]. However, further studies investigating its test-retest reliability are warranted. In the present study, 19% of the participants scored 0 (best possible score). Both FOGQ and FOGQsa consist of six items, where four items assess FOG severity and two items concern gait difficulties in general. Our results thus suggest that about 20% of the participants had neither gait difficulties nor FOG. In a study by Schrag et al., impaired gait was reported by 75% of people with a PD-duration of at least five years [31]. In another study, 21% out of 290 people with PD reported no gait problems [32]. The observed floor effect (i.e. percentage scoring 0) thus appears reasonable in the current targeted population. Reasonable floor and ceiling effects are a prerequisite for detection of differences [33]. Potential differences need also to exceed the measurement error in order to indicate a true change. The SEM observed in this study suggests that the change in scores need to be at least 1.7 in order to exceed the measurement error.

FOGQsa scores distinguished between fallers and non-fallers. Both fallers and those expressing a fear of falling had significantly higher FOGQsa scores than those not reporting these problems. This is not surprising since freezing is associated with falls [6,9-13]. Those who reported being unsteady while turning also had higher FOGQsa scores than those not reporting unsteadiness. In a study investigating 130 persons with PD, turning around was the factor most commonly identified to induce freezing [6]. Turning is also associated with falls [9,11,34], and falls associated with freezing are in fact more likely to result in an injury [9]. This underlines the importance of including an assessment of FOG when investigating falls in people with PD. Availability of a self-administered FOGQ may facilitate this in clinical practice.

When assessing FOG, Snijders et al. advocate combining physical examinations with more specific questions about FOG [3]. However, this is not possible when conducting postal surveys. FOGQ scores have also been shown to correlate only weakly with timed gait tests [25]. This indicates the need for using patient reported tools, especially since FOG is difficult to capture during a clinical or laboratory investigation [17,18]. The FOGQ is then preferable to using a single item approach, which is less reliable and less responsive to change [35]. Taken together, the present results speak in favor of using the FOGQsa when investigating FOG, falls and fear of falling in survey studies.

There are some methodological concerns which need to be taken into consideration. Demented patients were excluded, and the clinical sample was not randomly selected. This might affect the external validity of the present findings. The lack of maximum scores may suggest a bias towards less severe problems in the current sample. However, the highest observed score (22) is very close to the maximum score of 24, and the observed rate of people scoring zero on the FOGQsa is consistent with observations in previous studies (see above). Furthermore, validation of the FOGQsa (as well as the FOGQ) has been based on subjective data. Future studies may therefore consider adding ambulatory monitoring of FOG [36]. Finally, the fact that FOGQ includes two items concerning gait difficulties in general has been criticized, and these items were therefore omitted in a recent revised version of the FOGQ [37]. The revised version supplies a video-demonstration of FOG, but it is not self-administered. We have now initiated an adaptation in order to create a self-administered version of the revised version as well.

The Swedish FOGQsa described here can be obtained from the corresponding author. An English language version is under development and will also be obtainable from the corresponding author when available.

## Conclusions

In conclusion, we report the development and testing of the FOGQsa, a self administered version of the FOGQ. Its measurement properties are very similar to those previously reported for the FOGQ, suggesting that it is as reliable and valid as the original interview administered version. Importantly, FOGQsa scores do not differ from and correlate strongly with FOGQ scores. These findings suggest that no substantial information is lost by using the self administered version as compared to the clinician administered FOGQ. This facilitates the investigation of FOG in postal surveys and clinical practice. Further studies are warranted in order to scrutinize the measurement properties of the FOGQsa in more detail.

## Competing interests

The authors declare that they have no competing interests.

## Authors' contributions

MN conceived the study (including the development of the FOGQsa), participated in its design, data collection, conducted the statistical analyses, interpreted data and drafted the first manuscript.

G-M.H participated in designing the study, coordinated the survey study, participated in data collection and drafting the manuscript.

KW participated in designing the study, assisted in developing the FOGQsa and drafting the manuscript.

MM assisted in developing the FOGQsa and contributed to drafting the manuscript

LF participated in designing the study and drafting the manuscript.

PH conceived the study (including the development of the FOGQsa), participated in its design, data collection, interpretation of data and drafting the manuscript.

All authors read and approved the final manuscript.

## Pre-publication history

The pre-publication history for this paper can be accessed here:

http://www.biomedcentral.com/1471-2377/10/85/prepub
